# Host defense peptides for treatment of colorectal carcinoma – a comparative *in vitro* and *in vivo* analysis

**DOI:** 10.18632/oncotarget.2039

**Published:** 2014-05-29

**Authors:** Claudia Maletzki, Ulrike Klier, Samuel Marinkovic, Ernst Klar, Jörg Andrä, Michael Linnebacher

**Affiliations:** ^1^ Molecular Oncology and Immunotherapy, University of Rostock; ^2^ Department of General, Vascular, Thoracic and Transplantation Surgery, University of Rostock; ^3^ Department of Biotechnology, Hamburg University of Applied Sciences, Hamburg; Germany

**Keywords:** designer peptides, oncolytic therapy, individual tumor models

## Abstract

Host defense peptides (HDP) constitute effector molecules of the innate immune system. Besides acting against microbia and fungi, they exhibit broad and selective oncolytic activity. The underlying mechanism is at least partially attributable to elevated surface-exposed levels of phosphatidylserine (PS) on tumor targets. In this study, comprehensive analysis of NK-2-based derivatives (C7A, C7A-D21K, and C7A-Δ) was done on patient-derived ultra-low passage colorectal carcinoma (CRC) cell lines. Peptides were designed to improve antitumoral potential. Mellitin was used as positive control and a non-toxic peptide (NK11) served as negative control. Subsequently, effectiveness of local HDP application was determined in xenopatients.

Generally, CRC lines displayed a heterogeneous pattern of surface-exposed PS, which was usually below standard CRC cells. Of note, five out of seven cell lines were susceptible towards HDP-mediated lysis (lytic activity of peptides: C7A-D21K > C7A-Δ= C7A). Oncolytic activity correlated mostly with surface-exposed PS levels. Apoptosis as well as necrosis were involved in killing. In an *in vivo* experiment, substantial growth inhibition of HROC24 xenografts was observed after HDP therapy and, surprisingly, also after NK11 treatment.

These promising data underline the high potential of HDPs for oncolytic therapies and may provide a rationale for optimizing preclinical treatment schedules based on NK-2.

## INTRODUCTION

Antimicrobial peptides, also referred to as Host defense peptides (HDP), have originally been identified as an alternative weapon against bacterial infections. This is particularly important, as clinical handling of multi-resistant bacterial variants is increasingly difficult. These peptides represent an ancient yet very effective part of the innate immune system as first line of defense against invading bacteria. They are ubiquitous in nature and were identified in a variety of multi-cellular organisms including insects, amphibians and mammals [1,2]. HDPs exhibit a unique mode of action. Due to their cationic amphipathic nature they preferentially bind to negatively charged membranes [3-5] – a common characteristic of bacterial membranes, thus making them a perfect HDP target. HDPs incorporate into the membrane bilayers and form pores in targeted membranes leading to unsustainable cell homeostasis [6,7]. Additionally, HDPs penetrate the cytoplasm and interact with essential intracellular molecules. Unlike commonly used antibiotics, these cationic peptides directly interact with the target cells' lipid matrix instead of a specific enzyme or receptor.

Drug resistance, either intrinsic or acquired, is a growing problem in cancer therapy, as well. Although recent years have seen major treatment advances, cancer is still the third leading cause of death worldwide [8]. Surgery, chemotherapy, and radiation are the methods of choice in oncological management. The major drawback of the latter two is the missing selectivity between malignant and normal cells. Due to this unspecific toxicity most patients suffer from severe side effects. Additionally, there is growing evidence of multidrug resistant variants resulting for example from increased expression of multidrug resistance proteins. These proteins discharge antineoplastic drugs out of the cell and make them inefficient. This substantial problem emphasizes the necessity for developing new oncolytic agents.

**Table 1:** Clinical characteristics of patients and molecular data of the corresponding tumor

Tumor-ID	Age/Gender	Tumor location	TNM-Stage	Tumor type	Molecular type
HROC18	65/f	caecum	G2T2N0M0	primary adenocarcinoma	spStd
HROC24	98/m	colon ascendens	G2T2N0M0	primary adenocarcinoma	spMMR-D
HROC32	83/f	colon ascendens	G2T4N2M1	primary adenocarcinoma	spStd
HROC40	69/m	colon ascendens	G3T4N0M0	primary adenocarcinoma	CIMP-H
HROC60	71/m	colon ascendens	G2T2N0M0	primary adenocarcinoma	CIMP-H
HROC69	62/m	colon ascendens	G3T3NoMx	primary adenocarcinoma	spStd
HROC80	72/m	caecum	G2T3N2Mx	primary adenocarcinoma	spStd
HROC87	76/f	colon ascendens	G3T3N0M0	primary adenocarcinoma	spMMR-D
HROC107	81/f	colon ascendens	G3T3N0M0	primary adenocarcinoma	spMMR-D
HROC113	41/f	colon ascendens	G3T4N2Mx	primary adenocarcinoma	Lynch Syndrome

m – male, f – female, spStd – sporadic standard, spMMR-D – sporadic mismatch repair deficient, CIMP-H – CpG island methylator phenotype high, HNPCC – hereditary non-polyposis colorectal carcinoma

It has become apparent that HDPs are not only effective against bacteria. Impressive results from pilot studies identified selective killing of malignant, but not normal cells by different HDPs [9-11]. Physiologically, the outer membranes of mammal cells consist of zwitterionic phosphatidylcholine and sphingomyelin without any net charges. The negatively charged phosphatidylserine (PS) is usually a component of the inner cell membranes' leaflet. Under certain conditions, like loss of membrane asymmetry or cellular apoptosis, PS can be shifted to the cell surface [12]. In this case, PS renders cells vulnerable to lysis by cationic peptides like HDPs. Quite a number of tumor cells exhibit increased levels of surface-exposed PS [13,14]. This feature clearly distinguishes malignant from normal cells and provides a rationale for HDP-based tumor therapy [15,16]. For that reason, this study aimed at testing the oncolytic potential of NK-2-based HDPs [17] against patient-derived colorectal cancer (CRC) cell lines in order to pave the way for further treatment optimizations based on these peptides.

## RESULTS

### Peptide design

Peptides (C7A, C7A-D21K, C7A-Δ), and NK11 used in this study were based on NK-2 (Table 2 and [17]). The most basic modification entailed replacement of the non-functional sole Cys7 residue within the NK-2 sequence with an Ala residue (C7A). This substitution has been shown to improve anti-cancer cell activity of the lead structure NK-2 [17]. An enhancement of C7A's positive net charge was achieved by substituting Asp21 by a Lys residue (C7A-D21K). Moreover, peptide C7A was shortened by deletion of a stretch of four amino acid residues (including Asp21), resulting in peptide C7A-Δ. In addition, we used Melittin, the main lytic component of bee venom as a well-known reference compound, and NK11 as a non-toxic control peptide [21].

**Table 2:** Amino acid sequences of synthetic peptides used in this study

Peptide		Sequence^1^	Net charge^2^
	NK-2	KILRGVCKKIMRTFLRRISKDILTGKK	+10
#1	C7A	KILRGVAKKIMRTFLRRISKDILTGKK	+10
#2	C7A-D21K	KILRGVAKKIMRTFLRRISKKILTGKK	+12
#3	C7A-Δ	KILRGVAKKIMRTFLRR ILTGKK	+10
#4	NK11	KISKRILTGKK	+6
#5	Melittin	GIGAVLKVLTTGLPALISWIKRKRQQ	+6

1 all peptides were synthesized with an amidated C-terminus

2 The net charge of the peptides was calculated by subtracting the number of Asp residues (the only negatively charged amino acid residues present in the peptides) from all the positive charges (Lys, Arg and the peptide's N-terminus). Since the C-terminus of all peptides used was amidated, it did not bear a negative charge.

### Membrane intercalation of peptides monitored by FRET spectroscopy

**Figure 1:**
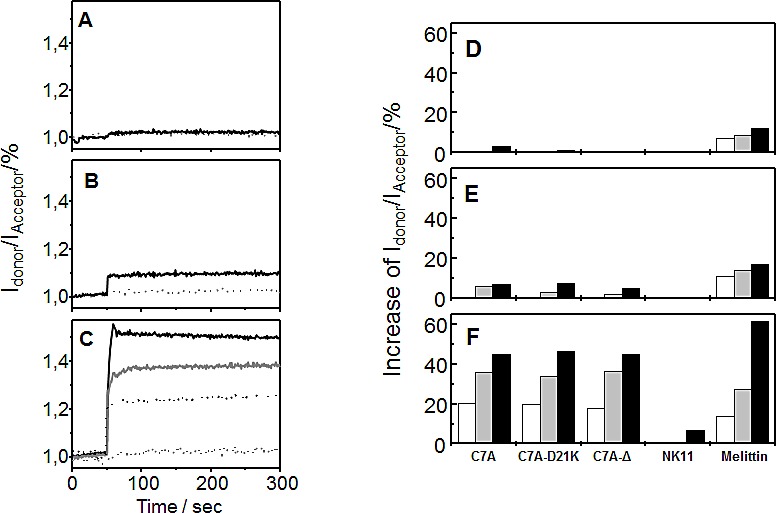
FRET spectroscopy. (A-C) Binding to and insertion of peptide C7A-D21K into phospholipid membranes (A) C7A-D21K was added at time point 50 s to liposomes (10 μM) consisting of pure PC, (B) a 90:10 molar mixture of PC:PS, and (C) of pure PS, each doubly doped with the fluorescently labeled FRET pair NBD-PE (donor) and rhodamine-PE (acceptor). Intercalation was monitored by measuring donor and acceptor fluorescence emission intensities. For better visualization only the quotient (I_Donor_/I_Acceptor_) is shown. Addition of the peptide solvent alone had no effect (dotted grey line). Peptide concentrations used: 0.2 μM (dotted black line, only in C), 0.4 μM (solid grey line, only in C), and 0.8 μM (solid black line). (D-F) Binding to and insertion of peptides at different concentrations into phospholipid membranes. (A) Peptides were added to liposomes consisting of pure PC, (B) a 90:10 molar mixture of PC:PS, and (C) of pure PS, each doubly doped with the fluorescently labeled phospholipid FRET pair NBD-PE (donor) and rhodamine-PE (acceptor). Intercalation was monitored by measuring donor and acceptor fluorescence intensities. The relative increase of the I_Donor_/I_Acceptor_ ratio 250 s after peptide addition is shown (Increase of I_Donor_/I_Acceptor_ (%) = (I_Donor_/I_Acceptor_ (peptide) - I_Donor_/I_Acceptor_ (control))*100). Peptide concentrations used: 0.2 μM (open bars), 0.4 μM (grey bars), and 0.8 μM (black bars).

FRET spectroscopy served as a sensitive tool to detect lipid dependence of the membrane interaction of antimicrobial peptides. For this, all peptides were added to liposomes consisting of zwitterionic PC alone, of a mixture of PC and of negatively charged PS, as well as of pure PS (Figure 1). All lipid vesicles were doped with donor and acceptor dyes, peptides were added, and the emission intensities of both dyes were monitored over time. An increase of the fluorescence intensity of the donor (I_Donor_) and a simultaneous decrease of the fluorescence intensity of the acceptor dye (I_Acceptor_), i.e. a reduced FRET efficacy, indicated an increase in the overall mean distance between labeled phospholipids, and corresponded to an insertion of peptides into the lipid bilayer. For clarity, the I_Donor_/I_Acceptor_ ratio is shown (Figure 1).

Insertion kinetics are shown representatively for peptide C7A-D21K (Figure 1A-C) and the maximum increase of I_Donor_/I_Acceptor_ is depicted for all peptides in figure 1D-F. In general, negatively charged PS triggers membrane intercalation of all peptides. With the exception of Melittin, no considerable intercalation was observed into pure PC liposomes. Upon addition of peptides to liposomes, we observed an immediate increase of the I_Donor_/I_Acceptor_ ratio, demonstrating a rapid interaction kinetic (Figure 1B,). Intercalation of the NK-2 derivatives C7A, C7A-D21K, and C7A-Δ was pronounced into pure PS bilayers (Figure 1C,) and slightly impaired into PC bilayers containing 10% PS (Figure 1B,). NK11 interaction was visible only with pure PS vesicles, but negligible with other liposomes.

### PS expression on tumor cells

As a first step towards identifying HDP-susceptible cells, level of surface-exposed PS was determined using our panel of low-passage CRC cell lines. For better estimating PS levels, amounts of AnnexinV bound to normal lymphocytes (PBL) and standard CRC cell lines (HCT116, SW48, TC71), and HDC114 were determined as well.

**Figure 2:**
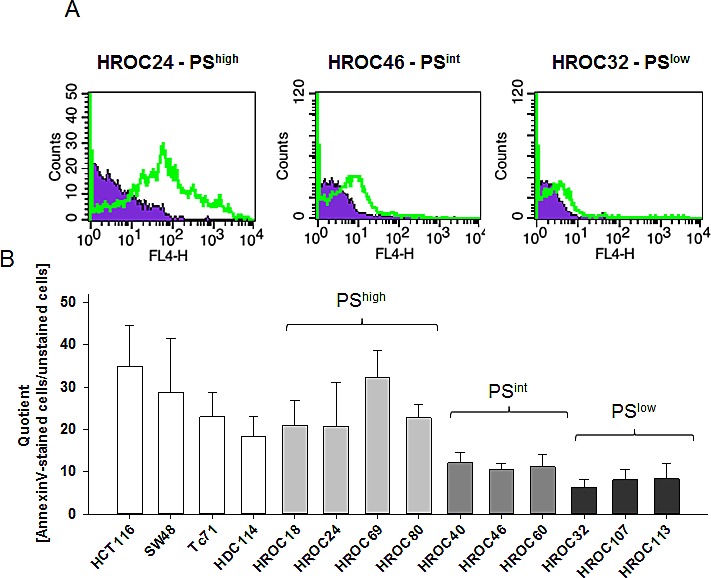
Flow cytometric analyses Surface-bound PS was detected on CRC cell lines following staining with APC-labeled AnnexinV. (A) Representative histograms of AnnexinV-stained tumor cells (thick green line) and corresponding unstained controls (filled purple histogram). (B) Amounts of surface-bound PS were quantified by calculating the quotient of the mean fluorescence intensities (MFI) from unstained cells versus AnnexinV-stained cells. Results show data of three independent experiments (mean ± SD).

Analysis revealed striking differences between individual cell lines, ranging from high (e.g. HROC69 and HROC80) to low (e.g. HROC32 and HROC107) surface-exposed PS. When comparing with established standard CRC lines, PS levels tended to be lower (Figure 2). However, fluorescence intensity was always higher than on normal lymphocytes that displayed PS at extremely low level (quotient ≤3; data not shown). Starting from these findings, CRC lines were classified as high, intermediate (int) or low PS-harboring cells (Figure 2).

### Response to HDPs according to PS-expression status

To prove growth inhibiting and cytotoxic activity of newly designed NK-2 analogues, tumor cells were exposed to increasing peptide concentrations for 1h and 24h, respectively. According to our classification, we included three PS^high^ (HCT116, HROC18, and HROC24), three PS^int^ (HROC40, HROC60, and HROC113), and two PS^low^ (HROC32 and HROC107) surface-exposed cell lines.

**Figure 3:**
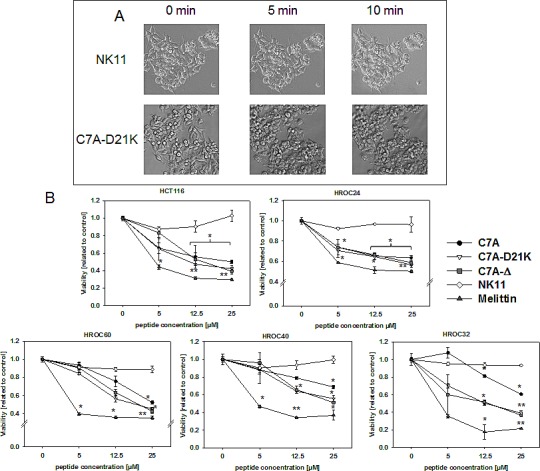
Short-term exposure of HDPs towards tumor cells (A) Representative pictures showing morphological changes in HROC24 cells upon HDP C7A-D21K exposure (25 μM). As a control, HROC24 cells were treated with NK11 (25 μM). (B) Quantitative analysis of direct cytotoxicity towards tumor cells. Tumor cells were treated for 1h with increasing HDP concentrations. Thereafter, viability was assessed using Calcein-AM staining. Remaining viable tumor cells were quantified in comparison to untreated controls, which were set to be =1. Results show data of three separate experiments. Values are given as the mean x-fold increase ± SD, *p<0.05 vs. NK11; **p<0.01 vs. NK11.

Short-term exposure revealed strong response towards all peptides, with, however, cell line specific susceptibility (Figure 3). Morphological changes even appeared after a 10 min incubation period (representative pictures for HROC24 exposed to peptides C7A and NK11, respectively, are displayed in Figure 3A). Unspecific killing was excluded by lack of NK11-mediated tumor cell lysis (Figure 3).

**Figure 4:**
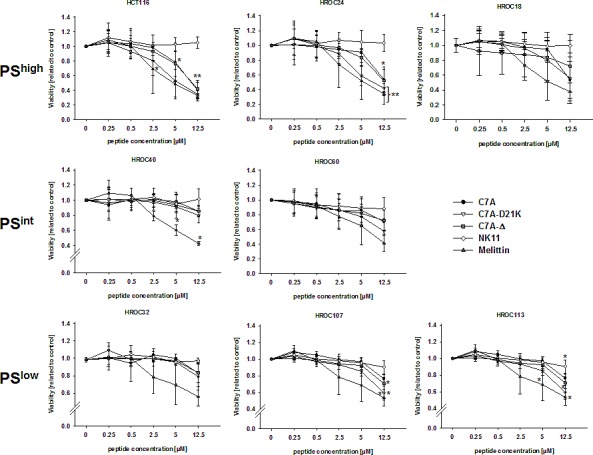
HDP-mediated cytotoxicity towards tumor cells Quantitative analysis of direct cytotoxicity towards tumor cells following a 24h incubation period with increasing HDP concentrations. Viability was assessed using Calcein-AM staining. Remaining viable tumor cells were quantified in comparison to untreated controls, which were set to be =1. Results show data of three separate experiments. Values are given as the mean x-fold increase ± SD. *p<0.05 vs. NK11; **p<0.01 vs. NK11.

Next, response after longer treatment schedules, i.e. 24h was examined. These analyses principally confirmed results from short-term exposure. HCT116 and HROC24 cells responded best; all tested HDPs exerted lytic activity towards these two cell lines (Figure 4). Of note, these peptides (C7A, C7A-D21K), and C7A-Δ were as effective as the reference compound Mellitin. HROC60 cells showed weaker though still noticeable vulnerability. In this cell line, obvious killing was obtained for C7A-D21K (>40% lysis vs. control). By contrast, HROC40 cells were relatively resistant towards HDP-induced lysis, even at high concentration (12.5 μM). When analyzing susceptibility of PS^low^ cell lines varying effects were obtained (Figure 4). Viability of HROC107 and HROC113 cells decreased dose-dependently. Of note, C7A-D21K proved to be most effective in killing these tumor cells. HROC32 cells did not respond well to HDPs. Overall, these observations are indicative for partial involvement of PS in the oncolytic action of HDPs.

### Predominant necrosis induction by HDPs

**Figure 5:**
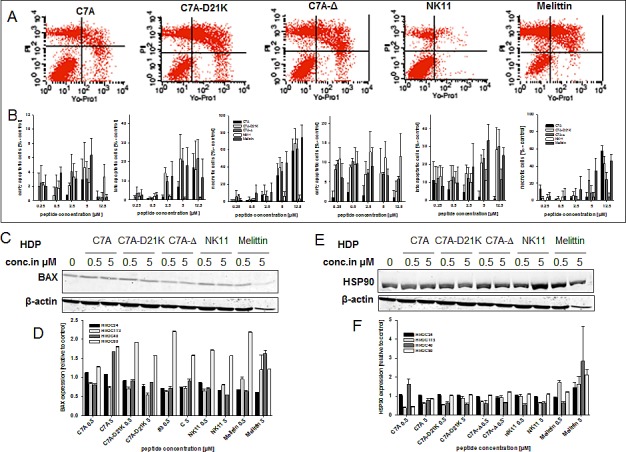
Apoptosis/necrosis assay and Western blot analysis (A, B) HROC18 and HROC24 cells were exposed to increasing HDP concentrations for a period of 24 hours. Tumor cells were stained with YO-PRO-1 for detecting early and late apoptotic tumor cells. Prior to flow cytometric analysis, PI was added to detect necrotic cells, as well. (A) Representative dot plots showing HDP-treated HROC24 cells at a concentration of 5 μM. Lower right quadrant: early apoptotic cells; upper right quadrant: late apoptotic cells; upper left quadrant: necrotic cells. (B) For cell death quantification, values of background cell death (=untreated controls) were subtracted from values of HDP-treated cells. Left panel: HROC24 cell; right panel: HROC18 cells. (C, E) Representative western blot results showing HDP-treated HROC24 cells (20 μg of total protein per lane). (D, F) Quantitative analysis of BAX and HSP90 expression in HDP-treated cells was done as described in material & methods. Expression levels of untreated cells were set to be =1 and x-fold increases of HDP-exposed cells were calculated. Results show data of three separate experiments. Values are given as mean ± SD.

To gain deeper inside into the type of cell death induced by HDPs, selected tumor cell lines (namely HROC24 and HROC18, both PS^high^) were subjected to flow cytometric apoptosis/necrosis analysis. Using this test, we observed predominantly necrosis in both cell lines. Early apoptotic cells (YO-PRO-1^+^/PI^−^) were found at all concentrations (0.25 - 2.5 μM). However, levels were always below 20% (Figure 5A,). Higher concentrations increased the number of late apoptotic (YO-PRO-1^+^/PI^+^) and necrotic (YO-PRO-1^−^/PI^+^) cells, respectively. Levels of viable HROC24 cells fell to 17% (C7A-D21K and C7A-Δ) and 25% (C7A). Here again, peptides C7A-D21K and C7A-Δ proved to be as effective in tumor killing as Melittin. HROC18 cells presented with marginally lower lysis. In this cell line, C7A-D21K was the most effective one and even slightly better than Melittin (74 vs. 71% total killing; Figure 5B). Peptides C7A and C7A-Δ killed up to 60% of cells.

Subsequent western blot analysis principally confirmed that necrosis is the primary reason for HDP-mediated cell death. Protein expression levels of Bax and HSP90 remained largely unchanged in HDP-responsive cell lines (e.g. HROC24, HROC40, and HROC113; representative blots for HROC24 are given in Figure 5C). The sole exception was seen for Melittin. Here, HSP90 expression was dose-dependently induced (Figure 5D). Cleaved PARP was not detectable at all.

### HDPs exert no hemolytic, but lymphotoxic effects *in vitro*

**Figure 6:**
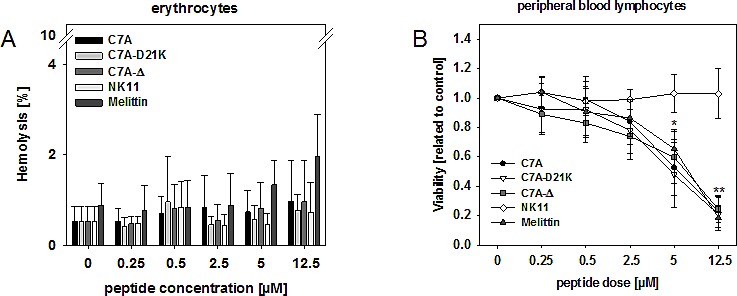
Hemolysis and lymphotoxicity assays (A) Hemolytic activity of HDPs was determined by hemoglobin release from whole blood cells after 120 min incubation. Negative controls were left untreated whereas positive controls (=maximum lysis) were treated with 1% SDS. Hemolytic activity was quantified as described in the material and methods section. (B) PBLs were incubated in the presence of increasing HDP concentrations for a period of 24 hours. Viability was analyzed using Calcein-AM and quantified in comparison to untreated controls, which were set to be =1. Results show data of six different healthy donors. Experiments were performed in triplicates. Values are given as the mean x-fold increase ± SD.

To examine if the observed lytic effects were tumor-specific, whole blood was cultured in the presence of increasing peptide concentrations for 24h. Experiments revealed absent or minimal hemolysis, with values below 20% even at high concentration (i.e. Melittin, dose: 12.5 μM, Figure 6A). In sharp contrast, all peptides reduced viability of lymphocytes in a dose-dependent manner (Figure 6B). Effects were most likely specific, since NK11 did not affect lymphocyte viability. Lymphotoxicity was independent from donor and gender.

### HDP-mediated tumor growth arrest *in vivo* is accompanied by increased tumor cell apoptosis

Finally, a proof of concept experiment was done *in vivo*. Female NMRI Foxn1^nu^ mice were inoculated with HROC24 cells, the cell line that showed highest vulnerability towards HDP-mediated killing *in vitro*. Mice with established xenografts received repetitive local injections of either peptide C7A or C7A-D21K. NK11 served as negative control. Melittin, which was applied as positive control *in vitro*, was excluded from this experiment due to its suspected high toxicity.

**Figure 7:**
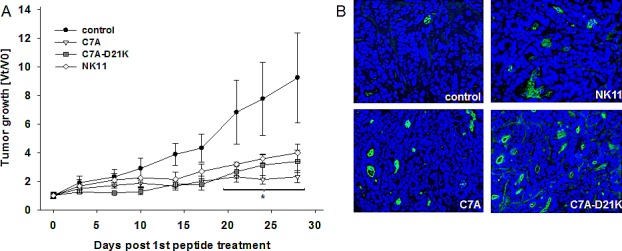
*In vivo* growth kinetics and tumor cell apoptosis of HROC24 tumors in NMRI Foxn1mice with or without local treatment (A) Therapy was performed by repetitive local application of HDPs C7A or C7A-D21K (1 mg/kg bw) every third day (a total of 9 injections) (n=5 per group). Tumor-carrying control animals received either equivalent volumes of peptide NK11 or saline (n=5 per group). Tumor volumes are given as x-fold increase vs. day 0 (V_t_/V_0_) (start of treatment) ± SD. *p<0.05 vs. PBS. (B) Representative immunofluorescence staining of apoptotic cells within HROC24 tumors. Cryopreserved tumor sections were stained with anti-M30 CytoDeath antibody, followed by anti-mouse IgG FITC antibody and nuclear DAPI as described in material and methods.

All treatment schedules were well tolerated by mice, with no adverse side effects, like weight loss, anemia, or ataxia. Application of peptide C7A demonstrated most effectiveness (Figure 7A). Tumors immediately reduced growing and macroscopically started to break up (in 4/5 cases). Of note, this arrest was evident until the end of experiments. A comparable initial delay was obtained following peptide C7A-D21K therapy (Figure 7B). HROC24 xenografts presented with strong growth retardation, though tumor growth was not completely impeded, especially at later time points (day 17). Opposed to C7A-treated tumors, no ulceration was observed. Finally, tumor sizes reached 3.4-fold increases (vs. PBS control: 10.3-fold). However, obtained effects were rather independent from specific NK-2 therapy, since control peptide NK11 induced tumor growth retardation as well. Though growth delay occurred to a lesser extent as compared to peptides C7A and C7A-D21K, there was still a significant effect (4-fold vs. PBS control: 10.3-fold, p<0.05). This finding was completely unforeseen since no antitumoral effects were found *in vitro*.

In line with the observed growth inhibition, all HDP-treated tumors showed marked increased levels of apoptotic tumor cells (Figure 7B). As given by positive M30 CytoDeath staining, apoptosis was most strongly induced in peptides C7A and C7A-D21K treated tumors, respectively. Tumors that had been exposed to peptide NK11 showed elevated cytokeratin 18 cleavage, as well. Besides, all HDP-treated tumors were considerably more necrotic than their untreated counterparts (data not shown).

## DISCUSSION

HDPs have gained much attention as alternatives to conventional chemotherapeutics; they bypass multidrug-resistance mechanisms and exert additive oncolytic effects in certain combinations [22, 23]. Due to the limited number of experimental preclinical studies, we here analyzed HDP action on a series of ultra-low passage, patient-derived CRC cell lines. These cells provide ideal models for testing novel drugs, since they closely resemble the original tumors' molecular and biological signature [19, 24]. HDPs applied in this study were based on NK-2, a porcine immune cell-derived peptide, whose selective killing of different human cancer lines is well established [10]. NK-2 derivatives exhibit reduced sensitivity towards oxidation, show improved antibacterial and –most importantly– oncolytic activity [17].

NK-2 associated cytotoxicity was shown to be at least partially attributable to preferential binding towards surface-exposed PS. In this study, varying levels of surface-bound PS were detected on our freshly established CRC lines, ranging from high (e.g. HROC24) to very low (e.g. HROC32) levels, close to normal lymphocytes. Amounts of surface-exposed PS were independent from (I) molecular CRC subtype, i.e. chromosomal/microsatellite instability or CpG-island and methylator phenotype, that have been found to impede drug response [25, 26]; (II) the corresponding mutational profile (i.e. K-ras, B-raf, p53, APC status); or (III) clinicopathological parameters (gender, age, TNM stage). Besides, there was no change over serial passages (at least from passage 10-50; data not shown), indicating that each tumor line harbors its own individual PS profile. Nonetheless, we occasionally observed differences between our patient-derived and “long-term” established standard cell lines, with tendency towards higher surface-exposed PS on the latter. On a basis of this initial finding, functional analyses were done for defining HDP-sensitivity and to test if the above mentioned mechanism also accounts for CRC lysis. Of note, all three newly designed NK-2 analogues (C7A, C7A-D2K1), and C7A-Δ exhibited antitumoral activities, even after short-term treatment schedules (1h). Although there was a trend towards higher susceptibility of cells displaying high PS surface-exposed levels (e.g. HROC24), vulnerability towards HDPs was more likely cell line and peptide specific. Two out of seven cell lines were completely resistant towards HDP-mediated lysis (i.e. HROC40^int^ and HROC32^low^). Generally, derivative C7A-D21K proved to be most effective in killing CRC lines. Hence, substituting Asp^21^ by a Lys residue yielded higher antitumoral potential. Peptide C7A-Δ, which is a shortened form of C7A-D21K, presented with killing potential, as well. By deleting four amino acids (including anionic Asp^21^ and cationic Lys^20^), the peptides' oncolytic activity was maintained. Moreover, these results provide evidence for the active domain to be within this region.

Overall, our findings indicate partial PS-dependent killing; yet, other mechanisms are likely to be involved as well. As a consequence of HDP-associated cell injury, apoptosis and necrosis play a role in cell death [10, 22, 27]. Quite in line, we here also observed both modes with a prevalence of necrosis. The rapid killing kinetic (<1h) of NK-2 based derivatives further supports this finding. This strong membranolytic effect should even make it difficult for tumor cells to develop resistance. This is of particular meaning, since acquired resistance to (targeted) cancer therapies is increasingly observed in the clinics and represents a major problem [28].

A rather unexpected observation of the current study was the massive cytotoxicity against normal lymphocytes. Although erythrocytes were not affected by HDPs, viability of lymphocytes dramatically decreased upon HDP-exposure. This is in sharp contrast to previous studies in which lymphotoxicity for NK-2 was designated to be low or absent [10]. However, cytotoxicity towards the vascular system has been demonstrated for some HDPs [22]. Cathelicidin (=LL-37), an antimicrobial protein that is produced by leukocytes, epithelial, and mucosal cells is cytotoxic to human oral squamous carcinoma cells, but also to human PBLs [29]. Comparable results were reported for BMAP-27 & BMAP-28. These HDPs cause apoptosis of leukemic cells, but also hemolysis of red blood cells [30]. Though this finding may limit *in vivo* applicability, we still wanted to address the question whether NK-2 based analogues have the potential to control tumor disease *in vivo*. So far, only few studies described successful *in vivo* application of oncolytic HDPs in general [16, 27, 31] and neither applied NK-2 and/or derivatives thereof. Here, immunocompromised nude mice were engrafted with HROC24 cells. This cell line was chosen on the basis of *in vitro* responsiveness and reliable engraftment [19]. A total number of nine intratumoral injections of peptide C7A or C7A-D21K resulted in a notable tumor growth delay; tumors even stopped growing for several days and remained significantly smaller than PBS-treated control tumors. This growth inhibition was accompanied by increased induction of cellular apoptosis. Of note, no adverse side effects, like hemolysis or lymphotoxicity, were found. These peptides could thus be identified as potent and well tolerable antitumoral compounds. This is particularly important since doses applied here were lower than most of the published ones for HDPs [27, 31]. Hence, tumor growth control might be the result of: (I) suppressed tumor cell proliferation (i.e. reduced Ki-67 expression); (II) direct tumor cell killing (most likely via necrosis); and (III) angiogenesis inhibition.

Contrary to our expectations, NK11 application also affected HROC24 tumor growth *in vivo*. NK11 is an 11-residue derivative that was applied as an inactive and thus nontoxic control. Nonetheless, HROC24 xenograft growth was significantly impaired by this protein. Additionally to the fact that prior studies on HDPs excluded control peptides [16, 27, 31, 32], several reasons might explain this observation: Firstly, the applied doses *in vitro* differed from those given *in vivo*. Secondly, NK11 was injected repetitively *in vivo*, while it was only once given to the cell culture. Thirdly, HDPs are known to be potent immune stimulators, especially of the innate immune system; hence unspecific immune activation is very likely.

We therefore propose the following model: local application of HDPs mediates rapid killing of tumor cells. However, the tumor microenvironment (including stromal cells) prevents parts of the tumor from lysis. Due to their natural behavior, HDPs enter the bloodstream and are recognized by the hosts' immune system, primary macrophages and NK cells. As a consequence, cells of the immune systems' innate arm infiltrate tumors. This proinflammatory local milieu helps controlling tumor growth transiently. Due to the lack of adaptive immune cells in nude mice, specific immune responses cannot be induced, preventing xenografted tumors from being completely eradicated. It is therefore conceivable that NK-2 and its derivatives exert substantially stronger antitumoral effects in immunocompetent hosts. Hence, oncolytic designer HDPs represent a very promising tool for inclusion into (chemotherapy-based) combinatorial treatment strategies. Due to the potentially rather unspecific mode of action, initial clinical studies should focus on intratumoral application.

## MATERIAL AND METHODS

### Tumor cell lines, lymphocyte preparation and culture media

CRC cell lines HROC18, HROC24, HROC32, HROC40, HROC57, HROC60, HROC69, HROC80, HROC87, HROC107, and HROC113 were established in our lab from patients subsequent to surgical resection. Molecular characterization was performed as described before [18,19]. Clinical characteristics of patients and molecular data of the corresponding tumor can be gathered from Table 1. The CRC cell line HCT116 (Kras^mut^, Braf^wt^) was originally obtained from the German collection of cell cultures (DSMZ; Braunschweig, Germany) and routinely cultured in our lab. For some experiments, established CRC lines SW48 (Kras^mut^, Braf^wt^), TC71, and HDC114 (both Kras^wt^, Braf^wt^) were included as well. Cells were maintained in full medium: DMEM/HamsF12 supplemented with 10% fetal calf serum (FCS), glutamine (2 mmol/L) and antibiotics (medium and supplements were purchased from PAA, Cölbe, Germany). Peripheral blood lymphocytes (PBL) were obtained from healthy volunteers following Ficoll density-gradient centrifugation (n=6).

### Synthetic Peptides

All peptides were synthesized with an amidated C terminus by the Fmoc solid-phase peptide synthesis technique on an automatic peptide synthesizer (model 433 A, Applied Biosystems; Darmstadt, Germany) and purified by HPLC as described [20]. Peptide stock solutions (1 mM) were prepared by solubilization of the purified and lyophilized peptides in 0.01% trifluoroacetic acid and were stored at -20°C.

### Lipids

Natural phospholipids L-α-phosphatidylcholine (PC) from hen egg and L-α-phosphatidylserine (PS) from porcine brain were purchased from Avanti Polar Lipids (Alabaster, AL, USA). Fluorescently labeled phospholipids N-(7-nitrobenz-2-oxa-1,3-diazol-4yl)-phosphatidylethanolamine (NBD-PE) and N-(lissamine rhodamine B sulfonyl)-phosphatidylethanolamine (rhodamine-PE) were from Molecular Probes (Eugene, OR, USA). Lipid stock solutions were prepared in chloroform.

### Förster resonance energy transfer (FRET) spectroscopy

Intercalation of peptides into liposome membranes was determined by FRET spectroscopy applied as a probe dilution assay. Liposomes were prepared by dissolving appropriate lipid mixtures (pure PC, a 90:10 molar mixture of PC:PS, as well as pure PS, each doped with 1% of the donor dye NBD-PE and 1% of the acceptor dye rhodamine-PE) in chloroform and drying them under a gentle stream of nitrogen. The resulting lipid film was dispersed in 20 mM HEPES, 150 mM NaCl, pH 7.4, sonicated for 30 min and subjected to 3-4 temperature cycles from 4 to 60°C with an incubation period of 30 min at each step. Lipid dispersions were stored at 4°C overnight before use. For the assay, donor fluorescence (NBD) was excited at 470 nm, and donor (NBD, ID at 531 nm) as well as acceptor (rhodamine, IA at 593 nm) emission intensities were monitored over time. An increase of the ratio of the donor fluorescence intensity to that of the acceptor fluorescence intensity (I_Donor_/I^Acceptor^) was interpreted as a membrane intercalation event. This ratio depends on the Förster efficiency, therefore a rising value means that the mean distance separation of donor and acceptor dyes is raising. The assay was performed at 37°C.

### AnnexinV staining of tumor cells and lymphocytes

AnnexinV staining was applied for detection of surface-exposed phosphatidylserine (PS) on tumor cells. Controls included lymphocytes from healthy volunteers. Cells (2 × 10^5^ cells/tube in PBS with Ca^2+^/Mg^2+^) were stained with APC (Allophycocyanin-) labeled AnnexinV (5 μl/tube, Immunotools, Friesoythe, Germany) for 20min at 4°C. Cells were washed, resuspended in 200 μl PBS and subjected to flow cytometric analysis, which was always performed on a FACSCalibur Flow Cytometer (BD Pharmingen, Heidelberg, Germany). Data analysis was performed from 20.000 events of each sample using CellQuest software (BD Pharmingen). Dying or dead cells were excluded by propidium iodide (PI). PI (1 mg/ml) was applied directly to cells prior to measuring. Amounts of surface-exposed PS were quantified by calculating the quotient of the mean fluorescence intensities (MFI) from unstained cells versus AnnexinV-stained cells.

### Cell viability staining

This assay is based on Calcein-acetoxymethylester (Calcein-AM; Invitrogen, Darmstadt, Germany) staining to detect viable cells within a culture. Tumor and normal (PBL) cells (1-2 × 10^4^ cells/well in triplicates) were seeded in 96-well plates and incubated overnight. Thereafter, peptides were added in increasing concentrations (ranging from 0.25 μM – 12.5 μM). Untreated cells served as living cell control. Medium was removed after 1h or 24h, respectively. Calcein-AM (4 mM) was added and incubated for 30 min (37°C, 5% CO_2_). Analysis was performed on a fluorescence multi-well plate reader (Tecan Infinite® M200, Crailsheim, Germany) at an excitation wavelength of 485 nm (emission 535 nm). For estimation of cell viability, the relative fluorescence intensities of Calcein-AM-stained non-treated cells (=live control) were set to be 1, and fluorescence intensities of samples were calculated. Data of at least three independent experiments each performed in duplicates are given.

### Apoptosis/necrosis assay

Cell death was quantified by YO-PRO-1 (Invitrogen) and PI double staining. Selected tumor cells (HROC24 and HROC18, 1 × 10^4^ cells/well) were seeded in 24-well plates and incubated overnight. Thereafter, peptides were added in increasing concentrations (ranging from 0.25 μM – 12.5 μM). Untreated cells served as living control. Following a 24-hour incubation period, supernatants were collected; cells were trypsinized and washed in PBS. Cells were stained with YO-PRO-1 (final concentration: 0.2 μM) for 20 min at room temperature. Prior to flow cytometric analysis, PI was added at a final concentration of 100 μg/ml. For cell death quantification, values of background cell death (=untreated controls) were subtracted from values of HDP-treated cells.

### Hemolysis assay

Hemolytic activity of HDPs was determined by hemoglobin release from whole blood cells after 120 min incubation. Briefly, whole blood of healthy donors (n=6) was seeded in 96-well plates and supplemented with HDPs at given concentrations (ranging from: 0.25 μM – 12.5 μM). Negative controls were left untreated and positive controls (=maximum lysis) were treated with 1% SDS. Following the incubation period, cell-free supernatants were transferred into a new 96-well plate and absorption was measured on a plate reader at 540 nm (reference wave length: 690 nm). Hemolytic activity was quantified according to the following formula:

$$\text{\% hemolysis}=(({\text{OD}}_{540\text{nm sample}}-{\text{OD}}_{540\text{nm buffer}})/{\text{OD}}_{540\text{nm max}}-{\text{OD}}_{540\text{nm buffer}})*100$$

### Western Blot analysis

Cellular protein extracts were obtained from HDP-treated and control cells following incubation with lysis buffer (1 M Tris, pH 7.5, 5 M sodium chloride, 0.25 M ethylenediaminetetraacetic acid, 10% (v/v) triton-x 100, 4% (v:v) sodium azide, 0.1 M phenylmethane-sulfonylfluoride, protease inhibitor cocktail (Roche, Mannheim, Germany)) on ice for 30 min. Cell lysates were centrifuged by 10.000×g for 10 min at 4°C. Protein concentration was determined using BCA Protein assay kit (Merck Calbiochem, Darmstadt, Germany) according to manufacturer's instructions. Proteins (20 μg) were separated on a 14% SDS-polyacrylamide gel and transferred to polyvinylidene fluoride membranes. Membranes were blocked for 1h with blocking buffer (Rockland, Gilbertsville, PA) prior to incubation with primary antibodies against Bax, cleaved PARP, HSP90, and β-actin (all New England Biolabs, Frankfurt am Main, Germany). IRDye® 800 CW- and IRDye® 680 CW-conjugated secondary antibodies (LI-COR-Biosciences, Bad Homburg, Germany) were applied for 30 min. Blots were scanned at a wavelength of 700 nm (for detecting IRDye® 680) and at a wavelength of 800 nm (for detecting IRDye® 800 CW) using an Odyssey® Infrared Imaging System (LI-COR-Biosciences). Signal integrated intensities were quantified applying the Odyssey® software version 3.16. Probes were normalized by calculating the ratio of the corresponding target protein to β-actin signal.

### *In vivo* tumor models and treatment regimen

Experiments were performed on female 8–10-week old NMRI Foxn1^nu^ mice (Charles River, Sulzfeld, Germany) weighting 18-20 g. All animals were fed standard laboratory chow and given free access to water. Trials were performed in accordance with the German legislation on protection of animals and the Guide for the Care and Use of Laboratory Animals (Institute of Laboratory Animal Resources, National Research Council; NIH Guide, vol.25, no.28, 1996). NMRI Foxn1^nu^ mice were challenged with 5 × 10^6^ HROC24 cells. Mice with established subcutaneous (s.c.) tumors received repetitive local injections of peptide C7A or C7A-D21K (1mg/kg bw, dissolved in 50 μl) every third day (a total of 9 injections) (n=5 per group). As control, tumor-carrying mice either received NK11 (1mg/kg bw, dissolved in 50 μl) or saline (50μl volume, n=5 per group). Tumor growth was controlled three times a week at time point of HDP injection and tumor volume was estimated according to the formula: V= width² * length * 0.52.

Tumor carrying mice were sacrificed at day 28 or when they became moribund before the tumor volume reached 2.000 mm³. At the end of each experiment, tumors, blood and spleens were collected from the animals of all groups for further analysis.

### Immunofluorescence

To study apoptotic cell death *in vivo*, 4 μm sections of HDP-treated and control tumors were mounted on poly-L-lysine-coated-slides. Following fixation in ice-cold methanol and blocking with 2% BSA (1h), cells were incubated with mouse anti-M30 CytoDeath antibody according to the manufacturer's instructions (Roche, Mannheim, Germany). A FITC-labeled anti-mouse IgG antibody was used as secondary antibody (Dako Envision Link, Hamburg, Germany). Cell nuclei were stained with DAPI and slides were embedded in mounting medium (Dako). Apoptotic cells were detected using a fluorescence microscope (Olympus, Münster, Germany).

### Statistical analysis

All values are expressed as mean ± SD. After proving the assumption of normality, differences between controls and treated cells (*in vitro*) and animals (*in vivo*) were determined by using the unpaired Student's *t*-test. If normality failed, the non-parametric Mann-Whitney *U*-Test was applied. The tests were performed by using Sigma-Stat 3.0 (Jandel Corp, San Rafael, CA). The criterion for significance was set to p<0.05.

## SUPPLEMENTARY FIGURE


