# Supplementary material for: Host defense peptides for treatment of colorectal carcinoma – a comparative in vitro and in vivo analysis
Source: Oncotarget. 2014 May 29;5(12):4467–79. doi: 10.18632/oncotarget.2039 (PMC4147338; doi:10.18632/oncotarget.2039)
Supplement: Supplementary file 1 [file oncotarget-05-4467-s001.pdf]

Host defense peptides for treatment of colorectal carcinoma – a comparative *in vitro* and *in vivo* analysis

Supplementary Material

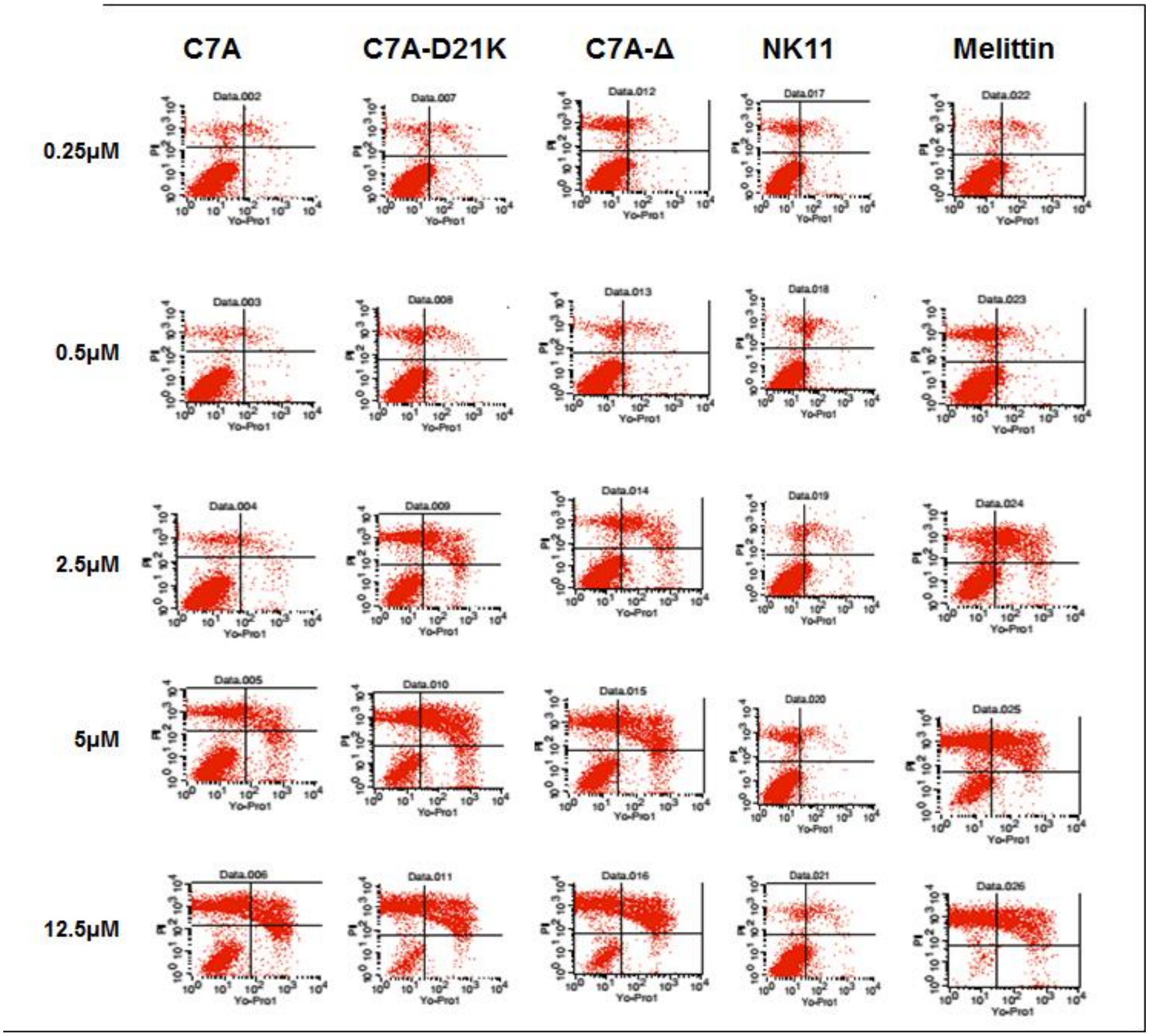

Supplemental Figure 1: Apoptosis/necrosis assay Representative dot plots showing HDP-treated HROC24 cells. HROC24 cells were exposed to increasing HDP concentrations for a period of 24 hours. Tumor cells were stained with YO-PRO-1 for detecting early and late apoptotic tumor cells. Prior to flow cytometric analysis, PI was added to detect necrotic cells, as well. Lower right quadrant: early apoptotic cells; upper right quadrant: late apoptotic cells; upper left quadrant: necrotic cells.
